# Environmental Cadmium: Arora et al. Respond

**DOI:** 10.1289/ehp.0901189R

**Published:** 2009-12

**Authors:** Manish Arora, Jennifer Weuve, Joel Schwartz, Robert O. Wright

**Affiliations:** Population Oral Health University of Sydney, Sydney, New South Wales, Australia, E-mail: marora@usyd.edu.au; Environmental and Occupational, Medicine and Epidemiology, Harvard School of Public Health, Boston, Massachusetts

We thank Guzzi et al. for their interest in our study on the association of environmental cadmium exposure and periodontal disease (Arora et al. 2009). There are a number of environmental sources of Cd in the U.S. population, with tobacco smoke being recognized as a major contributor (Paschal et al. 2000). In our study, we used creatinine- corrected urinary Cd concentrations to estimate long-term cumulative Cd exposure. This biomarker of Cd body burden encompasses an individual’s exposure to Cd from all sources; if dental restorative materials are indeed a source of Cd, then their contribution would also have been captured in our study.

That dental amalgams are the major source of Cd body burden has been questioned (Koh and Koh 2007), and further study is needed to determine the relative contribution of dental restorative materials to Cd exposure in the U.S. population. It is well recognized that the composition of dental amalgams and metal alloys used in dental restorations varies with type of restorative material and with the processes and standards of manufacture (Powers and Sakaguchi 2006). It therefore remains unclear whether any possible release of Cd from dental restorations would contribute significantly to the risk of periodontal disease.
